# IL-4Rα antagonist (Stapokibart) in refractory palmoplantar pustulosis: a case report and literature review of Th2-targeted therapies

**DOI:** 10.3389/fimmu.2026.1851685

**Published:** 2026-08-13

**Authors:** Yuqi Zhang, Yuexi He, Rao Leng, Wei Liu, Xiyuan Zhou, Jianing Yang

**Affiliations:** 1 Department of Dermatology, Sichuan Provincial People’s Hospital, University of Electronic Science and Technology of China, Chengdu, China; 2 School of Medicine, University of Electronic Science and Technology of China, Chengdu, Sichuan, China

**Keywords:** IL-4Rα monoclonal antibody, Palmoplantar Pustulosis (PPP), Stapokibart, STAT6 activation, Th2-related pathways

## Abstract

Traditionally associated with Th17-mediated pathways, palmoplantar pustulosis (PPP) is a persistent inflammatory dermatosis limited to the palms and soles. However, therapeutic responses to IL-17/IL-23 suppression are often limited, indicating significant immunological heterogeneity. There is growing evidence that type 2-associated immunological pathways may have a role in the persistence of disease in some patients. Herein, we report a 40-year-old woman with refractory PPP who had no personal or familial history of psoriasis or atopy. She was treated with stapokibart, an IL-4Rα monoclonal antibody that inhibits IL-4/IL-13 signaling, and experienced a rapid and long-lasting remission. Previous treatments, such as topical corticosteroids, retinoids, and Tripterygium wilfordii, were ineffective. Lesional skin exhibited moderate phosphorylated STAT6 positivity in scattered inflammatory infiltrates, independent of CD4+ T-cell presence, indicating engagement of canonical IL-4/IL-13 signaling at the tissue level, despite normal serum IgE levels and the lack of peripheral or tissue eosinophilia. The context-dependent clinical significance of Th2 pathway regulation in PPP is highlighted by a systematic literature review combined with our case. In non-atopic individuals, who frequently have noticeable pruritus despite unimpressive systemic atopic indicators, blockade of IL-4/IL-13 signaling has resulted in significant clinical relief. On the other hand, it has also been observed that blocking Th2-related pathways, such as IL-4/IL-13, IL-5, or IL-31, paradoxically causes PPP, highlighting the bidirectional and context-dependent significance of Th2 signaling in disease pathogenesis. Together with clinical characteristics like excessive pruritus, tissue-level Th2 activation may offer useful indicators to direct precision treatment in this diverse illness.

## Introduction

Palmoplantar pustulosis (PPP) is a long-term, recurrent inflammatory dermatosis that only affects the palms and soles. It is characterized by scaling, erythema, and recurrent sterile pustules, and it significantly lowers quality of life (1–4). The pathophysiology of PPP is still not well understood. PPP has always been categorized as a limited clinical variation within the spectrum of psoriasis (2, 5, 6). However, based on variations in genetic susceptibility, clinical history, and frequent connection with specific infections like tonsillitis, mounting evidence suggests that PPP is a nosological entity separate from generalized pustular psoriasis (GPP) (7–9).

In PPP, targeting the IL-17/IL-23 axis has shown therapeutic success. However, the idea that PPP has a unique profile is supported by the fact that clinical reactions are generally less severe than those seen in plaque-type psoriasis. Only a small percentage of patients were able to obtain deep or sustained clearance in randomized therapeutic trials including IL-17A and IL-23 inhibitors (10–12). Systemic Janus kinase inhibitors, on the other hand, have demonstrated effectiveness in biologic-refractory PPP, most likely by concurrently modulating several inflammatory pathways, including as IFN-γ and IL-36 signaling (13). All of these results point to the possibility that PPP pathogenesis is influenced by inflammatory factors other than the traditional IL-17/IL-23 axis.

Transcriptome analysis and new clinical data have suggested that type 2 immune pathways may play a part in PPP. Th2-related inflammation may contribute to disease manifestation in a subset of patients with treatment-refractory PPP, according to case reports that show clinical improvement after IL-4Rα inhibition, thereby decreasing IL-4/IL-13 signaling, most notably with dupilumab (14).

On the other hand, people taking dupilumab for atopic dermatitis have been documented to experience paradoxical induction of PPP or pustular psoriasis (15, 16). Transcriptome analysis have revealed immunological reprogramming in pustular psoriasis, with molecular characteristics similar to those seen in idiopathic PPP. This reprogramming is marked by a change from Th2 to Th17-dominant inflammation (15). Taken together, these disparate clinical outcomes strongly suggest that the Th2 axis’s function in PPP is context-dependent rather than homogeneous and may be impacted by genetic background, past treatment exposure, and baseline immunological traits.

Here, we demonstrate the first effective treatment of recalcitrant PPP in a patient without traditional atopic characteristics using stapokibart, a new high-affinity monoclonal antibody that targets IL-4Rα. Concurrently, we conducted a systematic literature search across PubMed, Embase, and Web of Science to comprehensively evaluate Th2-targeted therapies associated with either PPP induction or improvement.

## Case presentation

**Figure 1:**
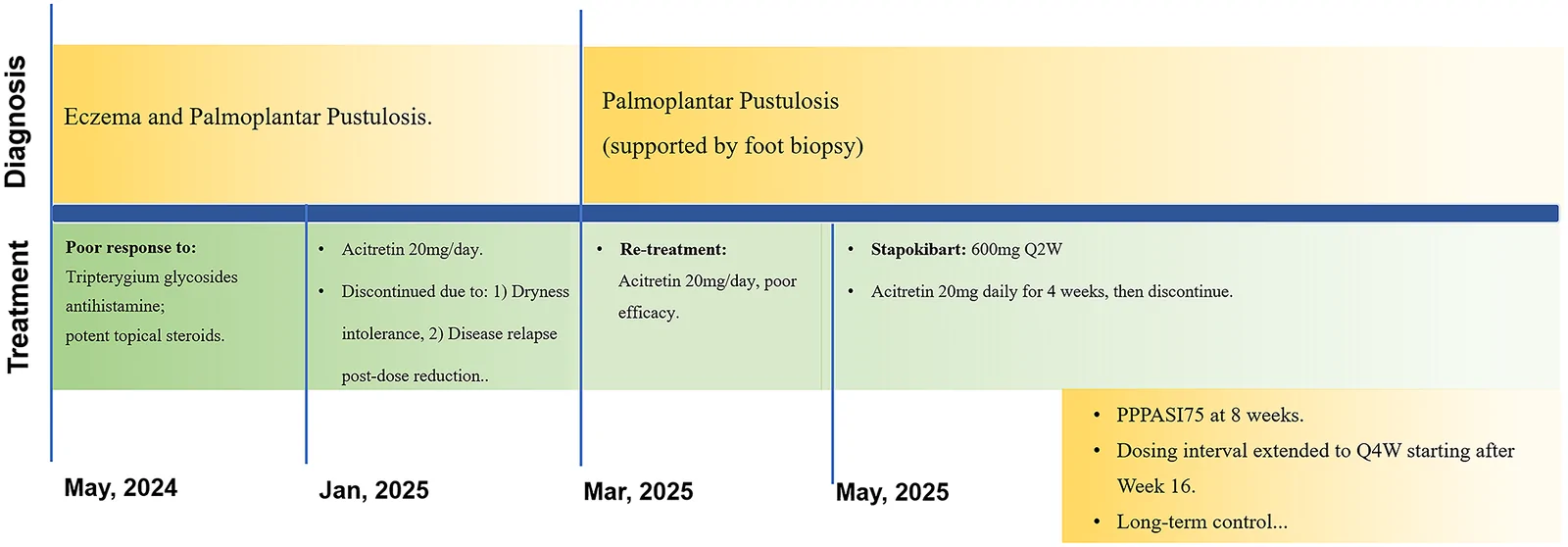
Timeline of treatments administered during the disease course.

A 40-year-old woman presented with a 10-month history of acute pruritus, sterile pustules on the palms and soles, and recurrent erythema. The patient denied having any family or personal history of atopy or psoriasis. She reported no history of cigarette smoking, denied dental focal infection or recent dental procedures, and had no clinical evidence of arthritis. After several negative mycological investigations of the palms and soles, she had previously been diagnosed with hand eczema and later diagnosed with palmoplantar pustulosis. For almost 12 weeks, she had undergone several therapies, including oral *Tripterygium wilfordii* glycosides, but there had been no discernible change. Although oral acitretin had been started on occasion, it had previously been stopped because of intolerance (specifically, cheilitis). Acitretin was reintroduced at a dose of 20 mg/day two months before presentation, however the condition was not adequately controlled. Additionally, concurrent topical treatments such as clobetasol propionate and halometasone are ineffective. **Figure 1** summarizes the course of treatment.

**Figure 2:**
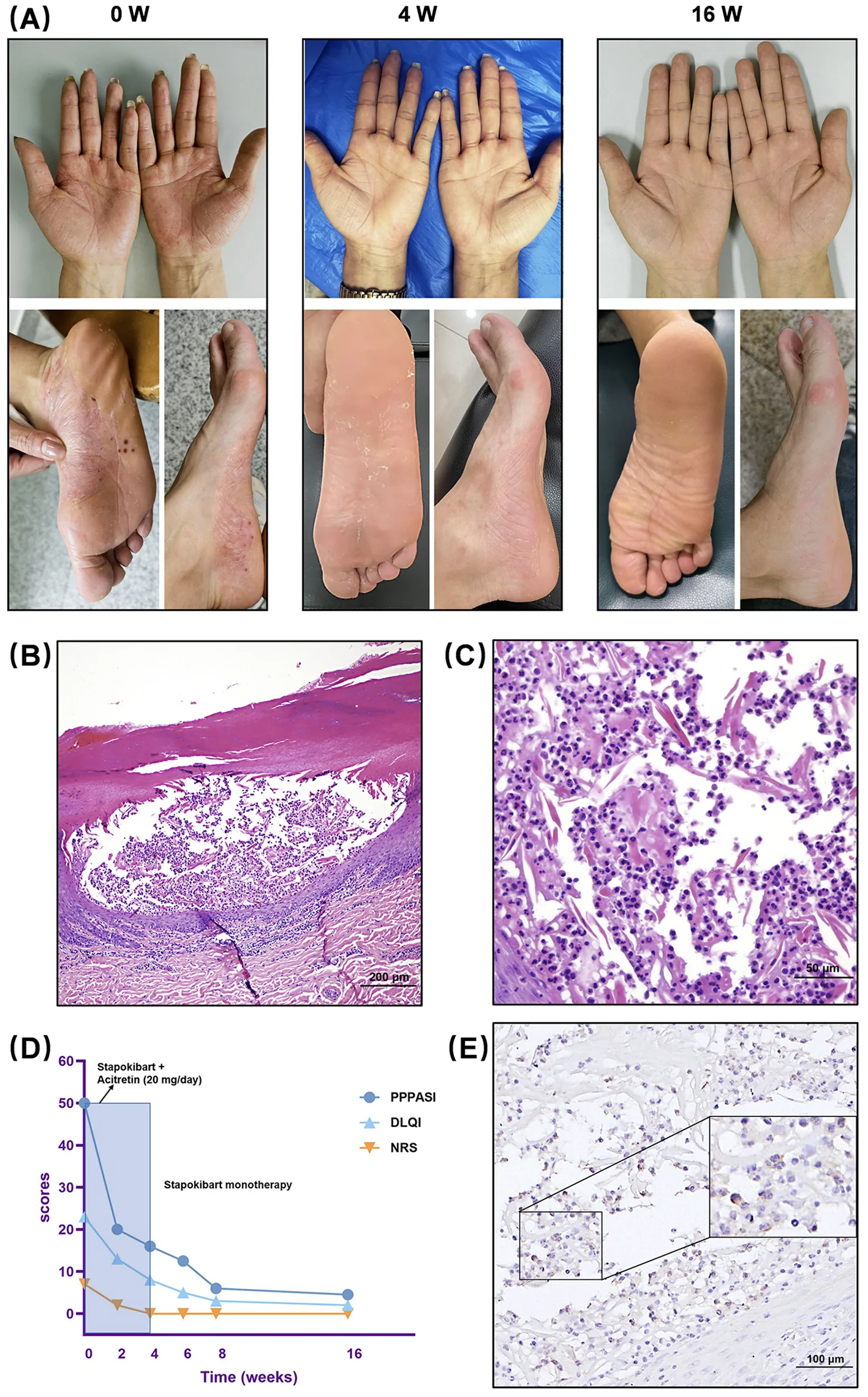
Clinical response, disease activity scores, histopathology, and immunohistochemical findings in a patient with PPP treated with stapokibart. **(A)** Typical clinical images of the palms and soles taken at baseline, week four, and week sixteen following the start of treatment. **(B)** Low-power hematoxylin and eosin (H&E) staining showing psoriasiform epidermal hyperplasia with intraepidermal pustule formation (scale bar: 200 μm). **(C)** High-power H&E staining revealing an intraepidermal pustule cavity densely packed with neutrophils (scale bar: 50 μm). **(D)** Longitudinal changes in Palmoplantar Pustulosis Area and Severity Index (PPPASI), Dermatology Life Quality Index (DLQI), and pruritus numerical rating scale (NRS) from baseline to week 16. **(E)** Immunohistochemical staining for phosphorylated STAT6 (p-STAT6) shows detectable nuclear positivity in scattered inflammatory cells within pustular areas (scale bar: 100 μm; inset: higher magnification).

Upon physical examination, symmetric erythematous plaques with sporadic deep-seated yellowish pustules were found on the palms and soles. The palms showed mild scaling, but the soles showed considerable hyperkeratotic scaling (**Figure 2A**). The Palmoplantar Pustulosis Area and Severity Index (PPPASI) score was 50, the Dermatology Life Quality Index (DLQI) score was 23, the Palmoplantar Pustulosis Physician’s Global Assessment (PPPGA) score was 3, and the pruritus numerical rating scale (NRS) score was 7 (**Figure 2D**).

Tests for liver and renal function as well as full blood counts, were all within normal ranges. Peripheral eosinophil counts and total serum IgE levels were not increased. A biopsy specimen taken from the plantar lesion showed psoriasiform epidermal hyperplasia with club-shaped rete ridges, partial decrease or absence of the granular layer, and hyperkeratosis with isolated parakeratosis. Neutrophil-dominated intraepidermal pustules were found in the top spinous layer. A unilocular intraepidermal pustule containing abundant neutrophils was observed within the upper spinous layer (**Figures 2B, C**). Immunohistochemical staining revealed moderate, focal nuclear positivity for p-STAT6 in scattered inflammatory cells (**Figure 2E**).

Based on the distinctive clinical presentation and histological findings, palmoplantar pustulosis was diagnosed. Based on the lack of plaque-type psoriasis, the chronic sterile pustular course, negative fungal investigations, and the distinctive histological findings, differential diagnoses such as palmoplantar pustular psoriasis, dyshidrotic eczema, and tinea manuum or pedis were taken into consideration and ruled out.

Stapokibart treatment was started following a detailed discussion with the patient and confirmation of the diagnosis. In addition to oral acitretin (20 mg/day), topical halometasone used twice daily, and frequent emollient usage, a loading dosage of 600 mg was given subcutaneously, followed by 300 mg every two weeks. The patient experienced significant alleviation from pruritus five days after starting medication (NRS score dropped to 2), and pustule production stopped. No new pustules or vesicles were seen at the 4-week follow-up visit, and erythema had significantly decreased (**Figure 2A**). The patient continued stapokibart monotherapy with only emollients when acitretin was stopped due to sensitivity after four weeks of combined therapy. The patient attained a PPPASI-75 response by week 8. At week 16, the pustules had completely resolved, leaving only a little amount of scaling on the soles and minor residual erythema on the palms (**Figure 2A**). **Figure 2D** shows that the PPPASI score dropped to 4.5, the DLQI to 2, and the NRS to 0. Throughout the course of treatment, no adverse events were documented. After then, the stapokibart dosage interval was increased to 300 mg every four weeks. The patient was still in prolonged remission and had not relapsed at the time of the most recent follow-up (week 30).

## Discussion

PPP has long held an unclear place in the spectrum of psoriatic diseases. Due to early immunological investigations showing systemic Th17 skewing and decreased regulatory T-cell compartments, especially in the Barber variety (variety B), which usually coexists with plaque psoriasis, it has historically been considered a localized variation of pustular psoriasis (7, 17). However, growing treatment evidence casts doubt on the suitability of a disease paradigm in PPP that is solely Th17-centric. When compared to the exceptional clearance rates seen in plaque psoriasis, suppression of the IL-17/IL-23 axis in PPP has produced much more variable and often modest outcomes, prompting a re-evaluation of its underlying immunopathology. Although IL-23p19 inhibitors have definitively proven superior to placebo, their clinical profiles differ substantially. Guselkumab demonstrates robust high-threshold responses in network meta-analyses, whereas risankizumab significantly improves only lower-threshold endpoints (11, 12, 18). Ustekinumab, which targets the shared p40 subunit rather than p19, has conversely failed to demonstrate superiority over placebo (19). Within the IL-17 class, network meta-analyses confirm overall efficacy despite variations among individual agents. Brodalumab has achieved substantial long-term high-threshold clearance, while secukinumab missed similar endpoints in certain randomized trials (20–23). Paradoxical PPP-like eruptions have occasionally been reported during IL-17 inhibition, but these rare events do not undermine the overall therapeutic value of this class (24). Collectively, such striking variability in treatment responses, even among agents targeting the identical pathway, underscores the underlying immunopathological heterogeneity of PPP. This complexity is further highlighted by the clinical efficacy of Janus kinase (JAK) inhibitors in biologic-refractory cases (13, 25–27). By broadly modulating multiple downstream signaling cascades rather than blocking a single cytokine, JAK inhibitors provide therapeutic benefits where targeted biologics fall short, strongly suggesting that factors beyond simple Th17/IL-23 pathway blockade dictate disease persistence.

Interest in non-canonical inflammatory axes other than Th17 signaling has increased as a result of these results. In this regard, several genetic and translational investigations have started to link type 2-associated immunological pathways to PPP. A genetic link with atopic dermatitis and relationships between PPP and polymorphisms in the IL4/IL13 locus have been found by genome-wide analysis, suggesting a common Th2-related vulnerability (28). Proteomic profiling has also shown that traditional Th2 chemokines, such as CCL11 and CCL13, are upregulated in PPP sera along with typical cytokine changes after IL-4Rα suppression (29). Our example showed STAT6 activation inside lesional inflammatory infiltrates, which is consistent with our systemic findings and provides tissue-level evidence of classical IL-4/IL-13 signaling engagement in a therapeutically sensitive PPP phenotype (30, 31).

To systematically evaluate the clinical outcomes of Th2-targeted treatments in PPP, we conducted a comprehensive literature search across PubMed, Embase, and Web of Science. The search strategy combined terms for the disease (‘palmoplantar pustulosis’ OR ‘PPP’) with keywords related to Th2-pathway targets and therapies (including ‘Th2’, ‘IL-4’, ‘IL-5’, ‘IL-13’, ‘IL-31’, ‘IgE’, ‘TSLP’, and ‘dupilumab’). Studies reporting clinical outcomes of these targeted therapies in PPP patients were included in our review. Our literature-based evaluation (**Table 1**) indicates the existence of a clinically significant subgroup of PPP in which Th2-related pathway modification may be linked to clinical benefit within this diverse landscape. Further evidence that is now available suggests that Th2 signaling may have bidirectional effects in PPP, either improving the disease or, in certain situations, inducing paradoxical disease (14, 16, 29, 32–36). The most consistently successful Th2-targeted approach to date among documented treatment responses is suppression of the IL-4/IL-13 pathway, most notably with dupilumab.

**Table 1:** Reported cases of palmoplantar pustulosis associated with Th2-targeted therapies.

Part A. Therapeutic use of Th2-targeted agents in PPP							
Author (Year), Region	Age / Sex	Disease duration (years)	Prior treatments	Th2-targeted therapy	Clinical immune background	Laboratory / histological findings	Clinical outcome
Present case, China	40 / F	0.8	AH, TWT, TC, AC	Stapokibart (anti-IL-4/13)	Pruritus; non-atopic; no psoriasis history	IgE normal; eosinophils normal	PPPASI-75 at week 8; rapid pruritus relief
Zheng (2024) (2025), China (14, 29)	32 / F	1	TC, AH, SEC	Dupilumab (anti-IL-4/13)	No personal or familial psoriasis	Significant elevation of serum IL-4 coupled with reduction in IL-17A	PPPASI-75 at week 16
	52 / F	0.5	TC, AH, AC				PPPASI-75 at week 6
	32 / F	10	TC, TVD				PPPASI-75 at week 8
	55 / F	10	TC, AC, AH				PPPASI-75 at week 5
	61 / M	1	TC, AH				PPPASI-75 at week 9
	39 / M	0.5	TC, AH				PPPASI-75 at week 12
	23 / F	8	TC, AC				PPPASI-75 at week 11
	60 / F	1	TC, TVD, AH				Fail
	44 / F	4	TC, TVD, AC, SEC				PPPASI-75 at week 8
	28 / F	0.5	TC, TVD, AH				PPPASI-75 at week 8
Smith (2023), USA (32)	60 / F	7	AC, TC, TVD, NB-UVB, APR, SEC	Dupilumab (anti-IL-4/13)	No family history of PPP or plaque psoriasis; pruritic & painful pustules	Histology: Dermis showed neutrophils, sparse lymphocytes and eosinophils	Clearance ≤1 mo; remission ~2 yrs; relapse off-drug; no AEs
Zheng (2022), China (33)	52 / M	1.5	TC, TVD, SEC	Dupilumab (anti-IL-4/13)	Non-atopic; foot pruritus & swelling	NR	PPPASI-75 at week 3; rapid pruritus relief
Yang (2025) China (34)	66 / F	0.5	TC, TVD, AH, MTX, TWT, PFL, CGT	Omalizumab (anti-IgE)	No family history of psoriasis; painful & itchy pustules	Total IgE 2979.9 IU/mL; FS-IgG4 (egg)↑; after 1-yr upadacitinib, IL-4/13/25/33 and TNF-α decreased, most prominently IL-25	Omalizumab: partial pruritus relief; switched to upadacitinib (JAKi) with rapid, complete pustule resolution
Part B. Paradoxical induction of PPP by Th2-targeted therapies							
Author (Year), Region	Age / Sex	Primary disease	Time to onset of PPP	Th2-targeted therapy	Clinical immune background	Laboratory / histological findings	Management & outcome
Nakamura (2025), Japan (38)	45 / F	AD with nodular prurigo	1 week	Nemolizumab (anti-IL-31)	NR	WBC: 12,400/μL; Eosinophilia (17%); high TARC (1742 pg/mL);ASO↑	Discontinued Nemolizumab; resolved with oral prednisolone
Park (2022), Korea (16)	17 / M	AD	2 months	Dupilumab (anti-IL-4/IL-13)	NR	NR	Dupilumab stopped and oral cyclosporine initiated with improvement
	24 / M	AD	2 months		AD (father)	NR	Dupilumab continued with lesions controlled by topical corticosteroids.
Artosi (2023), Italy (37)	59 / F	Severe asthma	6 months	Mepolizumab (anti-IL-5)	Father with psoriasis	Slightly elevated CRP (0.9 mg/dL); histology: Psoriasis lesions	Mepolizumab continued; resolved with topical calcipotriol/betamethasone

TC, topical corticosteroids; TVD, topical vitamin D derivatives; AC, acitretin; AH, antihistamine; SEC, secukinumab; CR, complete-response; NR, nonresponse; MTX, methotrexate; TWT, tripterygium wilfordii tablets; APR, apremilast; PFL, paeoniflorin; CGT, compound glycyrrhizin tablets; AD, atopic dermatitis.

None of the patients who showed significant improvement with IL-4Rα inhibition, including our case and those described by Zheng et al., had a familial history of psoriasis or concurrent psoriasis, and none had a personal history of atopic disease (14, 29, 32–34). These findings suggest that Th2-targeted treatment response in PPP is not limited to psoriatic or classical atopic backgrounds. When clinical descriptions were available, pruritus was the most frequently reported and noticeable complaint among treatment case studies (32–34).

As seen in our patient, this pruritic phenotype usually occurred without raised serum IgE levels or peripheral eosinophilia, indicating that traditional systemic atopic indicators may not accurately reflect Th2-associated activity in PPP. In this situation, even if regular laboratory data are unimpressive, persistent or excessive pruritus may prompt examination of Th2 pathway involvement. Histopathological results differed among reported instances, which is consistent with this clinical heterogeneity. Some Th2-responsive patients had cutaneous eosinophilic infiltration, but others, like our case and the instances Zheng et al. reported, had no discernible eosinophilia in their skin or blood. These results collectively imply that a Th2-associated PPP may manifest as a pauci-eosinophilic inflammatory pattern, wherein eosinophils are not always necessary for IL-4Rα blockade-induced clinical response. In our instance, tissue-level activation of Th2-associated signaling without overt T-cell enrichment was supported by immunohistochemistry, which also showed STAT6 phosphorylation in inflammatory cells without apparent CD4 staining. While STAT6 is canonically activated downstream of IL-4 and IL-13 via JAK-mediated phosphorylation (30), STAT6 phosphorylation alone does not definitively establish these cytokines as the sole upstream drivers. In our case, the absence of CD4+ T-cell infiltration alongside detectable p-STAT6 signal suggests that innate lymphoid cells, macrophages, or other non-T-cell sources may be the primary contributors (35, 36). Nevertheless, the convergence of tissue-level p-STAT6 detection, genetic evidence linking IL4/IL13 to PPP susceptibility, and proteomic demonstration of serum Th2 signatures in PPP patients collectively supports the involvement of canonical Th2 signaling in a disease subset (28, 29).

In individuals without psoriasis, however, paradoxical activation of PPP has been observed following Th2-targeted therapy such as suppression of the IL-4/IL-13, IL-31, or IL-5 pathways (16, 37, 38). This duality emphasizes how Th2 regulation in PPP is context-dependent. Notably, reports of paradoxical occurrences and therapeutic responses have mostly come from Asian communities. This trend is consistent with known ethnic disparities in PPP epidemiology and immunogenetic background, including a decreased overlap with plaque psoriasis (1, 9, 39). It may also be partially due to reporting bias. Ethnicity should be viewed cautiously as a potential modifying factor rather than a determinant due to the small number of reported cases.

Rather than a permanent atopic diathesis, the activation of Th2 pathways in PPP seems to reflect dynamic immunological plasticity. A fragmented immunological pattern in PPP has been identified by transcriptomic and single-cell studies. This pattern is defined by Th17 polarization in circulating memory CD4^+^ T cells and Th2-skewed gene expression in lesional skin. *In vivo* Th17–Th2 lineage plasticity in PPP is directly demonstrated by the detection of GATA3^+^/CD161^+^ dual-positive T cells in both peripheral blood and lesional tissue (40). Crucially, this flexibility is context-dependent and reciprocal. When baseline immune tone favors these pathways, IL-4Rα blockade may trigger a shift toward Th17/IL-36–dominant inflammation, resulting in molecular features more similar to pustular psoriasis than plaque psoriasis, according to transcriptomic profiling of dupilumab-induced psoriasiform eruptions (15). All of these findings point to IL-4 and IL-13 playing a modulatory function in preserving inflammatory homeostasis, which may have an impact on keratinocyte-associated IL-36 responses. The known link between PPP and persistent localized infections may further influence the tendency for such immunological reprogramming. The overexpression of its ligand B7h in lesional skin and the increased expression of the co-stimulatory protein ICOS on tonsillar CD4^+^ T cells indicate that infection-driven immune activation may start local cytokine circuits that continue after the inciting stimulus (39). It also offers a plausible connection between localized infection, immunological co-stimulation, and downstream T-cell reprogramming because B7h expression is induced by IFN-γ and TNF-α, cytokines that are routinely increased in PPP (41–43). These results provide a unifying framework for both therapeutic responses and paradoxical reactions seen with Th2-targeted treatments, supporting a conceptual model in which PPP reflects an immunologically heterogeneous illness defined by fluctuating Th17, Th2, and IL-36 inflammatory setpoints.

This study has several limitations. First, based on a single case and a small number of published reports, it is impossible to draw firm conclusions about a unified Th2-related immunophenotype in PPP, given the disease’s significant immunological heterogeneity. Furthermore, our characterization of the Th2-associated phenotype was constrained by the lack of serum TARC measurements and the inability to clearly identify eccrine sweat duct involvement (acrosyringium), a recognized histopathological hallmark of PPP (44), in the available biopsy sections. Despite these limitations, to the best of our knowledge, this is the first documented clinical use of stapokibart, a high-affinity monoclonal antibody targeting IL-4Rα, in PPP. The rapid and persistent response observed in this non-atopic patient supports the idea that IL-4/IL-13 signaling may contribute to disease maintenance in a subset of PPP patients, independent of typical atopic propensity. Preclinical evidence showing stapokibart’s greater IL-4Rα binding affinity than dupilumab provides a possible, though unconfirmed, explanation for the variation in Th2-targeted treatments (45). This notion is similar to findings in psoriasis treatments, where variations in IL-17 inhibitor receptor-binding characteristics have been linked to variations in clinical efficacy. However, the significance of such pharmacological differences in PPP is still debatable due to the lack of head-to-head comparison research.

## Conclusion

Our results offer validity to the idea that immunologically diverse manifestations of palmoplantar pustulosis exist, and that Th2-associated pathways may play a role in disease activity apart from traditional atopic predisposition. Our non-atopic case’s positive reaction to IL-4Rα inhibition emphasizes the potential benefits of an endotype-informed therapeutic approach and emphasizes the necessity of further research using tissue-level biomarkers, including p-STAT6 expression, to improve precision therapy approaches in PPP.

## Data Availability

The original contributions presented in the study are included in the article/**Supplementary Material**. Further inquiries can be directed to the corresponding author.
